# m^6^A mRNA methylation initiated by METTL3 directly promotes YAP translation and increases YAP activity by regulating the MALAT1-miR-1914-3p-YAP axis to induce NSCLC drug resistance and metastasis

**DOI:** 10.1186/s13045-021-01048-8

**Published:** 2021-02-23

**Authors:** Dan Jin, Jiwei Guo, Yan Wu, Jing Du, Lijuan Yang, Xiaohong Wang, Weihua Di, Baoguang Hu, Jiajia An, Lingqun Kong, Lei Pan, Guoming Su

**Affiliations:** 1 Clinical Medical Laboratory, Binzhou Medical University Hospital, Binzhou, 256603 People’s Republic of China; 2 Cancer Research Institute, Binzhou Medical University Hospital, Binzhou, 256603 People’s Republic of China; 3 Department of Thyroid and Breast Surgery, Binzhou Medical University Hospital, Binzhou, 256603 People’s Republic of China; 4 Department of Pain, Binzhou Medical University Hospital, Binzhou, 256603 People’s Republic of China; 5 Department of Gastrointestinal Surgery, Binzhou Medical University Hospital, Binzhou, 256603 People’s Republic of China; 6 Department of Clinical Laboratory, Binzhou Medical University Hospital, Binzhou, 256603 People’s Republic of China; 7 Department of Hepatobiliary Surgery, Binzhou Medical University Hospital, Binzhou, 256603 People’s Republic of China; 8 Department of Respiratory and Critical Care Medicine, Binzhou Medical University Hospital, Binzhou, 256603 People’s Republic of China; 9 Department of Nursing, Binzhou Polytechnic University, Binzhou, 256603 People’s Republic of China

## Editor’s Note

Concerns have been raised about the integrity of the data reported in this article [1]. This is currently being investigated. Further editorial action may be taken as appropriate once the investigation into the concerns is complete and all parties have been given an opportunity to respond in full.
